# Clinical factors associated with a conservative gait pattern in older male veterans with diabetes

**DOI:** 10.1186/1757-1146-2-11

**Published:** 2009-04-23

**Authors:** James S Wrobel, Ryan T Crews, John E Connolly

**Affiliations:** 1 Center for Lower Extremity Ambulatory Research at Scholl College of Podiatric Medicine, Rosalind Franklin University of Medicine and Science, North Chicago, Illinois, USA; 2 VAM&ROC White River Junction, Vermont, USA

## Abstract

**Background:**

Patients with diabetes and peripheral neuropathy are at higher risk for falls. People with diabetes sometimes adopt a more conservative gait pattern with decreased walking speed, widened base, and increased double support time. The purpose of this study was to use a multivariate approach to describe this conservative gait pattern.

**Methods:**

Male veterans (mean age = 67 years; SD = 9.8; range 37–86) with diabetes (n = 152) participated in this study from July 2000 to May 2001 at the Veterans Affairs Medical Center, White River Junction, VT. Various demographic, clinical, static mobility, and plantar pressure measures were collected. Conservative gait pattern was defined by visual gait analysis as failure to demonstrate a heel-to-toe gait during the propulsive phase of gait.

**Results:**

Patients with the conservative gait pattern had lower walking speed and decreased stride length compared to normal gait. (0.68 m/s v. 0.91 m/s, *p *< 0.001; 1.04 m v. 1.24 m, *p *< 0.001) Age, monofilament insensitivity, and Romberg's sign were significantly higher; and ankle dorsiflexion was significantly lower in the conservative gait pattern group. In the multivariate analysis, walking speed, age, ankle dorsiflexion, and callus were retained in the final model describing 36% of the variance. With the inclusion of ankle dorsiflexion in the model, monofilament insensitivity was no longer an independent predictor.

**Conclusion:**

Our multivariate investigation of conservative gait in diabetes patients suggests that walking speed, advanced age, limited ankle dorsiflexion, and callus describe this condition more so than clinical measures of neuropathy.

## Background

Gait alteration in patients with diabetes has been described [1-3]. Patients with diabetes and peripheral neuropathy (DMPN) exhibit gait instability [4,5]. While this may appear trivial to the treating clinician, unsteadiness in gait demonstrated the strongest association with depressive symptoms in a study by Vileikyte and colleagues [6]. Chamberlin and colleagues identified fearful walkers from a Modified Falls Efficacy Scale. They found fearful walkers demonstrated a slower walking speed, shorter stride length, and longer double support time than walkers not identified as fearful [7]. Courtemanche and colleagues observed similar findings in DMPN patients. They found prolonged reaction times leading the authors to conclude that there are increased attentional demands with more conservative gait patterns suggesting lack of proprioception affecting control of gait [1]. Yavuzer and colleagues conducted a cross-sectional study of patients with DMPN (n = 20), diabetes (n = 26), and age-gender-BMI matched control patients (n = 20). They described patients with diabetes having slower gait, shorter steps, limited knee and ankle mobility, and lower plantar flexion moment and power than the control group. These differences were not significant for the DMPN group. Neuropathic patients were defined by electrophysiological testing and it is unclear to what degree this definition is associated with more coarse clinical definitions using monofilaments or vibratory perception threshold testing. The duration of diabetes was similar between the groups at 19 and 15 years. They also found that increased HbA1c and F-wave distal latency were significantly associated with decreased ankle mobility, peak plantar flexion moment and power [3].

While intuition suggests patients with diabetes adopt a more conservative gait pattern to make them feel more stable, they remain at higher risk for falls. Although most falls produce no serious injury, between 5% and 10% of community-dwelling fallers do sustain a serious injury with many failing to recover to their pre-injury level of function [8]. In a prospective study of 139 elderly patients in a long-term care facility, Maurer and colleagues looked at falls in multiple domains. These included clinical diagnoses, medications, orthostatic blood pressure change, gait, balance, mental status, well being, activities of daily living, affect, behavior, range of motion, and communication. In the multivariate model, diabetes, gait, and balance remained as significant and independent predictors [9]. Other case-control and cohort studies have described similar findings using multivariate analysis [9,10].

While patients with diabetes may adopt this more conservative gait pattern, we are not aware of any studies that looked at individual clinical attributes in a multivariate model within this specific population. The advantage of a multivariate approach is to control for other measured confounding variables, such as age and neuropathy status. The purpose of this study is to use a multivariate approach to describe this conservative gait pattern.

## Methods

### General design and study population

This study took place from July 2000 to May 2001 at the Veterans Affairs Medical and Regional Office Center, White River Junction, VT. The exact methods have been previously described and are overviewed below [11,12]. Patients were eligible if they were taking an oral hypoglycemic agent or insulin for diabetes and had no current foot ulceration. Patients with active foot and ankle injury, or history of ablative or elective foot surgery were also excluded. Participants signed an informed consent approved by the Committee for Protection of Human Subjects.

### Clinical examination

One examiner and the principal investigator underwent training prior to the inception of the study in order to assure standardization of examination techniques with previously published methods. Age, diabetes duration, smoking status, height, weight, HgbA1c within the past six months was collected prior to the examination. Pedal pulses were palpated and patients with the absence of one or more pulses were considered to have arterial insufficiency[13] Sensitivity to monofilament was determined using a 10 gram monofilament. The patient was insensate if they were unable to detect one or more of the following plantar sites, 1st metatarsal-phalangeal joint (MPJ), 5th MPJ, or hallux [14]. Available dorsiflexion at the ankle was measured as previously described [15]. Briefly, the patient was measured in the supine position with the knee on the frontal plane. The ankle was dorsiflexed maximally with the subtalar joint in a neutral position by palpation. The goniometer was aligned with the lateral column of the foot and lateral lower fibula. Available dorsiflexion at the 1st MPJ was measured passively with the patient standing in a relaxed posture. End range of motion in the dorsiflexed position was felt to be a more informative measure due to current theory in sagittal plane mechanics of the foot [16-18]. The inter-rater reliability, as measured by the intraclass correlation coefficient was 0.71 for the ankle and 0.95 for the 1^st ^MPJ [12].

In a weight-bearing state, the presence of a bunion deformity, hammer toes, foot architecture, and postural sway were determined. Bunion deformity was present if there was abducted great toe position with prominent medial eminence to the 1st MPJ. A hammer toe was defined as a contracted toe requiring a dorsiflexion force to move the digit. Foot architecture, Romberg's test, and joint position sense were performed as previously described [19,20]. The presence of a forefoot weight bearing callus was determined. Plantar forefoot fat pad atrophy was defined as a plurality of prominent metatarsal heads readily palpable on the plantar surface of the foot.

An apropulsive gait was defined by visual gait analysis where a patient failed to demonstrate a heel-to-toe gait during the propulsive phase of gait. While the inter-rater reliability of visual gait analysis has been questioned, a study of 20 patients using the observational gait scale, the investigators found moderate to substantial reliability[21] for heel rise with weighted kappas ranging from 0.47 – 0.78 (intra-rater) and 0.43 – 0.62 (inter-rater) [22]. The reliability of describing the push-off in gait after stroke was also described as ranging from moderately-high to high in physical therapists. The intraclass correlation coefficient ranged from 0.76 for inter-rater reliability to 0.89 for intra-rater reliability [23]. Walking speed was assessed by measuring the time taken to walk a 10 metre distance following a 3 metre pre-distance to assure constant velocity. Stride length was determined by measuring the distance a foot travels from initial heel contact to heel contact for the next stride of the same foot using a tape measure on the floor. The average of three trials was taken and the patient was coached to walk at their regular walking speed.

### Plantar pressure measurement

Mean dynamic foot pressures were measured using the F-Scan mat system, software version 4.12F (Tekscan, Boston, MA). Patients were studied using 4-inch stockinette for stockings and without shoes. Calluses were debrided prior to measurement. The mat was calibrated to the patient's weight and the sampling frequency was set at 50 Hz. Maximum peak plantar pressures for the entire foot were obtained using the average of three mid-gait foot steps.

### Statistical analysis

This is secondary analysis of an existing data set. The unit of analysis was the foot rather than the individual. Since the observations were not entirely independent, a generalized linear model was created using sandwich robust variance estimator and assuming Poisson errors and a log link to estimate relative risk for dichotomous errors. The dependent variable was binary, with 1 depicting the conservative or apropulsive gait pattern and 0 denoting normal propulsive gait. In the first part of the analysis, univariate analysis used a chi squared test with Fisher's Exact test for dichotomous data and one-way analysis of variance for continuous data. The multivariate model was built using a forward stepwise logistic regression with the criterion for removal being a p-value > 0.1. Of the 152 patients, 40 patients had the conservative gait pattern. Based on this, we nominated 4 *a priori *covariates for our regression model. These included age, neuropathy status, and dorsiflexion at the ankle and 1^st ^MPJ.

## Results

Patients with the conservative gait pattern had lower walking speed and decreased stride length compared to normal gait. (0.68 m/s v. 0.91 m/s, *p *< 0.001; 1.04 m v. 1.24 m, *p *< 0.001) Table 1 describes the descriptive characteristics of our population and univariate analysis. Age, neuropathy, and Romberg's sign were significantly higher; and ankle dorsiflexion was significantly lower in the conservative gait pattern group. Presence of peripheral arterial disease (as measured by palpable pulses) and callus approached significance. Table 2 describes the multivariate analysis where walking speed, age, ankle dorsiflexion, and callus were retained in the final model (Model 1). This model described about 36% of the variance around the conservative gait strategy. In a stepwise fashion, walking speed and age described 24.8% and 4.7% of the variance respectively. Ankle dorsiflexion and callus described 3.5% and 2.8% of the variance respectively.

**Table 1:** Descriptive characteristics (values are means ± (SD) unless otherwise stated)

	**Conservative Gait**	**Normal Gait**	**p-value**
N	40	264	
Age (yrs)	73.1 (7.64)	66.2(9.83)	0.00
Insulin (% yes)	36	28	0.35
HbA1c (%)	7.89 (1.37)	7.64(1.69)	0.29
DM Duration (yrs)	9.5 (4.75)	10.1(10.38)	0.73
Smoking History(% yes)	92	82	0.11
Height (mean inches)	68.5(0.93)	68.4(2.82)	0.95
Weight (mean lbs)	212(43.95)	211(42.04)	0.85
1st MPJ ROM (degrees)	12.1(2.72)	14.2(7.66)	0.13
Bunion deformity (% yes)	18	17	1.00
Hammer toe (% yes)	51	37	0.11
Foot type (% yes)			
• normal	46	52	
• pronated	26	23	
• supinated	28	25	
Romberg's sign (% yes)	26	12	0.04
Non-palpable pulse (% yes)	59	43	0.06
Insensitivity to 10 gram monofilament (% yes)	46	27	0.02
Absent joint position sense (% yes)	5	2.6	0.35
Ankle DF (degrees)	3.6(2.07)	5.6(2.93)	0.01
Callus (% yes)	49	36	0.08
Fat pad atrophy (% yes)	31.4	47.1	0.12
Stride length (metres)	1.04(0.09)	1.24(0.17)	0.00
Walking speed (metres/second)	0.68 (0.08)	0.91(0.14)	0.00
Peak Pressure (kg/cm^2^)	3.81(0.73)	3.87(0.87)	0.75

**Table 2:** Multivariate analysis

**Multivariate Analysis**	**Risk Ratio**	**95% CI**	**p-value**	**Pseudo R^2^**
**Model 1**				
Walking speed Age in yrs.	0.00 1.09	0.00 to 0.00 1.04 to 1.15	0.00 0.00	
Callus Ankle DF (degrees)	3.43 0.86	1.38 to 8.54 0.79 to 0.96	0.01 0.01	0.36
				
**Model 2**				
Walking speed Age in yrs.	0.00 1.09	0.00 to 0.00 1.04 to 1.15	0.00 0.00	
Callus Ankle DF (degrees)	3.38 0.86	1.35 to 8.44 0.77 to 0.97	0.01 0.01	
Insensitivity to 10 gram monofilament (% yes)	1.15	0.47 to 2.83	0.75	0.36

## Discussion

As far as we know, this is the first published study to use a multivariate approach to study the conservative gait pattern in patients with diabetes. The prevalence of conservative gait in our cohort of elderly diabetic veterans was quite high at 26%. This compares favorably with the results of one study that found 23% of neuropathic patients reporting unsteadiness [6]. Another study found fearful walkers comprised 24% of their sample of community dwelling older adults [7].

The univariate analysis described age, neuropathy, Romberg's sign, callus, absent pulse, walking speed, ankle and 1^st ^MPJ dorsiflexion as being associated with the conservative gait pattern. Thinking that neuropathy would lead to an increased fear of falling and subsequently dispose neuropathic individuals to fearful walking, we were surprised that neuropathy was not retained in the multivariate model. We also tried Romberg's sign and absent joint position sense in place of neuropathy thinking that this represented advanced clinical neuropathy. This also was not retained in the final model. The findings are consistent with Yavuzer and colleagues where they did not see any difference between the patients with diabetes and diabetes with neuropathy [3]. These findings are also supported in part by the neuropathy findings of Mueller and colleagues; however, the unloading differences in conservative gait patterns are not found in our work [24]. Additionally, our approach addressed suggestions by the invited commentaries to Mueller et al. that patients without neuropathy and a population of patients that may not have been affected by treatment of foot ulcers be included [24].

Our study has a number of potential limitations. The cross-sectional design and secondary analyses make causal attribution problematic. While the present study is larger than many studies assessing applied biomechanics in patients with diabetes, it is still a select population of predominately male Veterans visiting foot clinics thus potentially limiting generalizability. Effectively, this was a blinded study as examiners were unaware that the conservative gait strategy approach was going to be used in a later analysis. Our neuropathy definitions were also coarse including testing only for monofilament sensitivity, great toe position sense, and Romberg's sign. One could also question the clinical significance of a two degree restriction in statically measured ankle dorsiflexion that was statistically significant. While our inter-rater reliability of this measure was moderate, other authors have described mean absolute differences of two degrees [25]. Other authors have also questioned the role of static measures versus dynamic measures with walking [12].

There are potential clinical implications of the study. Debridement of callus and potential exercise training in this population[26,27] could be investigated regarding their roles in conservative gait strategy. Limited ankle joint dorsiflexion could also be investigated dynamically to observe if this passive limitation persists, whereby the forward momentum of the tibia is restricted [28].

## Conclusion

Our multivariate investigation of conservative gait in diabetes patients suggests that walking speed, advanced age, limited ankle dorsiflexion, and callus describe this condition more so than clinical measures of neuropathy. The clinical implications of this work should be investigated further.

## Competing interests

The authors disclose no potential conflicts of interest including employment, consultancies, stock ownership, honoraria, paid expert testimony, and patent applications/registrations.

## Authors' contributions

JSW was the primary investigator and contributed to the specific aims, study design, patient examination, statistical analysis, and writing. JEC contributed to the specific aims, study design, and writing. RC contributed to the statistical analysis, interpretation of the results, and writing.
